# Cells Co-Producing Insulin and Glucagon in Congenital Hyperinsulinism

**DOI:** 10.3390/life16010018

**Published:** 2025-12-22

**Authors:** Yuliya Krivova, Alexandra Proshchina, Dmitry Otlyga, Diliara Gubaeva, Maria Melikyan, Sergey Saveliev

**Affiliations:** 1 Laboratory of Nervous System Development, Avtsyn Research Institute of Human Morphology of FSBSI “Petrovsky National Research Centre of Surgery”, Tsurupi Street, 3, 117418 Moscow, Russia; proshchina@yandex.ru (A.P.); otlyga@bk.ru (D.O.); brainmicroscopy@yandex.ru (S.S.); 2 National Medical Research Centre of Endocrinology; Dm.Ulyanova St., 11, 117292 Moscow, Russia; gubaevadn@gmail.com (D.G.); melikian.maria@gmail.com (M.M.)

**Keywords:** congenital hyperinsulinism, human pancreas, insulin, glucagon, PDX1

## Abstract

Alterations of pancreatic islet cell phenotypes are well established in diabetic conditions and considered to be one of the possible causes of insulin deficiency. However, there is limited information about alterations of islet cell phenotypes in opposite metabolic conditions such as hypoglycemia in infants with congenital hyperinsulinism (CHI). Surgical biopsies of the pancreas from six infants with diffuse CHI and five infants with focal CHI were examined using double immunofluorescence with antibodies against insulin, glucagon and the key transcriptional factor responsible for β-cell differentiation and maturation—PDX1. The phenotypes of cells within the pancreatic islets in diffuse CHI and within the focus in focal CHI were compared to those in unaltered pancreatic islets located outside the focus. In diffuse CHI, the proportion of bi-hormonal insulin+/glucagon+ cells was increased. Additionally, an increase in the proportion of insulin+ cells lacking PDX1 was observed in diffuse CHI and within the focus. It can be assumed that alterations of the phenotype of β-cells may occur under hypoglycemic conditions, but the role of islet cell plasticity in infants with CHI remains to be established.

## 1. Introduction

Congenital hyperinsulinism (CHI) is a heterogenous group of disorders characterized by insulin hypersecretion from β-cells [1,2]. Although CHI is rare (with an incidence of approximately 1:50,000 to 1:28,389, but up to 1:2500 in populations with high consanguinity rates), it is the most common cause of persistent hypoglycemia in neonates, infants, and children [3,4].

The causes of CHI are quite varied. To date, about 10 monogenic forms and several syndromic forms of CHI are known [1,5], although the molecular causes remain unknown in 21–54% of cases [6,7]. The etiology of monogenic forms of CHI is currently well understood. CHI is caused by disease-causing variants in genes belonging to three functional categories: channels; metabolic enzymes; and transcription factors that are central to beta-cell identity and proliferation [1]. Impairment of any of these functions leads to excessive insulin secretion and has significant clinical implications for the choice of treatment.

The choice of therapy is also significantly influenced by the histological form of CHI. Two main forms can be distinguished: the focal form (focal CHI) and the diffuse form (diffuse CHI) [8,9,10,11,12,13]. Focal CHI is characterized by a focal proliferation of atypical pancreatic islets, while the surrounding tissue remains relatively normal. In contrast, diffuse CHI involves abnormalities in pancreatic islets throughout the pancreas [14]. Additionally, a less frequent atypical form has been described, characterized by the presence of hyperplastic islets in several adjacent pancreatic lobes [10,12,13].

Although the role of β-cells in the pathogenesis of CHI is well studied, their plasticity and interactions with other cell types within the pancreatic islets are still not fully understood. Several studies have described signs of δ-cell immaturity in both focal and diffuse CHI, along with a reduced number of somatostatin-positive δ-cells, which normally suppress insulin secretion [13,15]. One study has also shown increased expression of NeuroD1, a transcription factor regulating endocrine cell differentiation, in pancreatic tissue from infants and children with CHI [13].

Experimental studies have demonstrated that hyperglycemia can induce β-cell dedifferentiation into immature progenitor-like cells or transdifferentiation into α-cells [15,16,17]. Reduced insulin gene expression and downregulation of β-cell–specific transcription factors such as PDX1, Nkx6.1, and PAX6 have been observed in rats after near-total pancreatectomy [16]. Similar findings, including β-cell degranulation, increased expression of immaturity markers (Neurogenin3, Oct4, Nanog, L-Myc) and decreased expression of β-cell transcription factors (PDX1, Nkx6.1, MafA), have been reported in several animal models of diabetes [17,18,19,20]. Some of these dedifferentiated β-cells may subsequently begin to express glucagon and other α-cell–specific markers, giving rise to bi-hormonal cells [17,19].

Morphological evidence from human pancreatic samples further supports the occurrence of β-cell dedifferentiation and transdifferentiation in diabetes. Cells co-expressing β-cell markers (insulin, Nkx6.1, PDX1, PC1/3, GLUT1) together with α-cell markers (glucagon, ARX, GLP-1, GC), in various combinations, have been identified in individuals with both type 1 [21,22] and type 2 diabetes [23,24,25]. The loss of β-cell identity through dedifferentiation and transdifferentiation is now recognized as a significant mechanism contributing to the decline in β-cell mass in diabetes [17,18,19,20]. β-cell dedifferentiation and transdifferentiation appear to be at least partially reversible, and β-cell mass can be restored following dietary or pharmacological normalization of glycemia [17,18,20], which represents a promising therapeutic avenue for diabetes. However, the mechanisms underlying β-cell plasticity remain uncertain.

Metabolic conditions in CHI are essentially the opposite of those in diabetes. The most common and severe forms of CHI are linked to inactivating mutations in genes encoding the subunits of the pancreatic β-cell ATP-sensitive potassium (KATP) channel (ABCC8, KCNJ11) [1]. In diffuse CHI, recessive or compound heterozygous mutations in ABCC8 or KCNJ11 affect all β-cells, leading to a complete loss of KATP channel function [1]. In focal CHI, a paternal monoallelic recessive mutation in ABCC8 or KCNJ11, followed by a somatic loss of heterozygosity of the 11p15 maternal allele in a restricted pancreatic region, results in the formation of focal adenomatous hyperplasia with structurally absent or non-functional KATP channels within the focus [1,14]. Non-functional KATP channels cause persistent plasma membrane depolarization, uncontrolled calcium influx, and constitutive, glucose-independent exocytosis of insulin granules [26]. This genetically driven insulin hypersecretion could contribute to β-cell degranulation in CHI. Histopathological findings in both focal and diffuse CHI indicate relatively weak staining for insulin in affected β-cells, confirming low insulin storage due to impaired insulin release [10]. Therefore, analyzing islet cell phenotypes in CHI may offer valuable insights into the physiological and pathological roles of β-cell plasticity.

In the present study, we evaluated the distribution of bi-hormonal insulin+/glucagon+ cells and key β-cell specific transcription factor – PDX1 in focal CHI (both within the focus and normal tissue) and diffuse CHI.

## 2. Materials and Methods

The study was conducted on pancreatic biopsies collected from five cases of focal CHI during lesion resection and six cases of pharmacoresistant diffuse CHI undergoing elective subtotal pancreatectomy (Table 1). CHI was diagnosed based on fasting test results, specifically persistently elevated insulin levels (>2.0 μU/mL) in the setting of hypoketotic hypoglycemia (plasma glucose < 3.0 mmol/L, ketonemia < 1.0 mmol/L).

Pharmacoresistance was defined clinically by the persistent inability to maintain euglycemia despite treatment with diazoxide and/or octreotide, high glucose infusion requirements, and supportive findings from molecular genetic testing and ^18^F-DOPA PET/CT imaging. The decisions to proceed to surgical treatment were made by an experienced multidisciplinary team after thorough case-by-case assessment and only when pancreatectomy was deemed absolutely necessary.

The histological form of CHI was initially suggested by molecular genetic testing and ^18^F-DOPA PET/CT and subsequently confirmed by histological examination of the surgical specimens. Informed consent was obtained from the legal representative of each patient. The study was approved by the Ethics Committee of the National Medical Research Center for Endocrinology (protocol No. 18 (9), 11 October 2017).

Pancreatic samples were fixed in 10% buffered formalin (pH 7.0–7.4; BioVitrum, Saint Petersburg, Russia). The samples were then dehydrated in graded isopropyl alcohol solutions, embedded in paraffin, and sectioned into 4-μm-thick sections. Sections were mounted onto Superfrost slides (Thermo Fisher Scientific Inc., Fremont, CA, USA) and stored at 4 °C.

Four out of the five patients with focal CHI (cases 1–4) had presumably healthy pancreatic tissue located outside the focal lesion. These samples, containing unaltered pancreatic islets, were used as a comparison group (Table 1). In one case of focal CHI, the available tissue sections contained only the lesion, with no adjacent unaltered pancreatic regions. Pancreatic specimens were divided into three groups: (1) focal CHI, histologically unaltered islets (adjacent tissue); (2) focal CHI, lesional tissue (focus); and (3) diffuse CHI.

Double immunofluorescent labeling was applied to analyze the distribution of cells containing insulin, glucagon, and PDX1 within the pancreas. The primary and secondary antibodies used, along with their dilutions, are listed in Table 2. For immunofluorescent labeling, sections were dewaxed, rehydrated, and subjected to antigen retrieval by microwaving in 10 mM citric acid buffer, pH 6.0 (Dia-M, Moscow, Russia) for 10 min, followed by cooling for 20 min. The sections were then incubated with a blocking solution consisting of 5% normal goat serum (Jackson ImmunoResearch Laboratories Inc., West Grove, PA, USA) in Tris-buffered saline with 0.1% Tween 20 (TBST; Thermo Fisher Scientific Inc., Fremont, CA, USA) for 30 min at room temperature. Primary antibodies were diluted in 1% normal goat serum in TBST and incubated at 4 °C for 12 h. Secondary antibodies were diluted in TBST and incubated at room temperature for 2 h. Sections were coverslipped using EverBrite™ mounting medium with DAPI (Biotium, Fremont, CA, USA). Negative control sections, in which primary antibodies were replaced with 5% normal goat serum in TBST, were included in every immunostaining run for each case.

Preparations were examined using an ADF U300 microscope equipped with a fluorescence module, digital microscopy camera Ultra09, and ADF Image capture software, version x64, 4.11.21522.20221011 (ADF Microscopes, Ningbo, Nanjing, China). All measurements were conducted using the ADF Image capture software.

The distribution of insulin-, glucagon-, and bi-hormonal insulin/glucagon-containing cells was assessed in tissue sections double-stained with mouse monoclonal anti-insulin (Thermo Fisher Scientific Inc., Cat.# MS-1379-P) and rabbit polyclonal anti-glucagon (Thermo Fisher Scientific Inc., Cat.# PA5-13442) antibodies. We captured at least ten images at ×400 magnification for each tissue type per patient: unaltered islets from unaffected tissue in four focal CHI cases, lesion areas with endocrine cells from focal CHI, and pancreatic islets from diffuse CHI. In focal lesion, the islet margin (regions of interests in which endocrine cells were quantified) was defined as the border between stained endocrine cell areas and adjacent unstained regions. For each islet (region of interest in focal lesion), we measured the islet area, the total number of DAPI-positive nuclei, and the number of cells of each specific type (insulin+, glucagon+, insulin+/glucagon+). We then calculated the cell density as the number of DAPI-positive nuclei per µm^2^ of islet area and determined the proportion of each cell type (insulin+, glucagon+, and insulin+/glucagon+) relative to the total number of DAPI-positive nuclei within the islet.

The distribution of PDX1 was analyzed on sections double-stained with the following combinations of antibodies: mouse monoclonal anti-insulin (Thermo Fisher Scientific Inc., Cat.# MS-1379-P) and rabbit polyclonal anti-PDX1 (Epitomics, Cat.# AC-0131C) antibodies; mouse monoclonal anti-glucagon (Sigma, Cat.# G2654) and rabbit polyclonal anti-PDX1 (Epitomics, Cat.# AC-0131C) antibodies. For each patient and each tissue type, ten islets from insulin+PDX1 double-stained sections and ten islets from glucagon+PDX1 double-stained sections were imaged. In insulin+PDX1 images, we counted the total number of insulin-positive cells, insulin-positive/PDX1-positive cells, and insulin-positive/PDX1-negative cells, and calculated the proportions of insulin+/PDX1+ and insulin+/PDX1− cells among all insulin+ cells. Similarly, in glucagon+PDX1 images, we counted total glucagon-positive cells, glucagon-positive/PDX1-positive cells, glucagon-positive/PDX1-negative cells, and calculated the proportions of glucagon+/PDX1+ and glucagon+/PDX1− cells among all glucagon+ cells.

Statistical analysis was conducted using Statistica 10 (StatSoft Inc., Tulsa, OK, USA). Data are presented as medians (Me) with the 25th (Q1) and 75th (Q3) percentiles. We applied the nonparametric Kruskal–Wallis ANOVA test, followed by post hoc multiple comparisons of mean ranks for all pairs of groups as the data did not follow a normal distribution. *p*-values < 0.05 were considered statistically significant.

## 3. Results

### 3.1. Co-Localization of Insulin and Glucagon in Diffuse and Focal CHI

**Figure 1:**
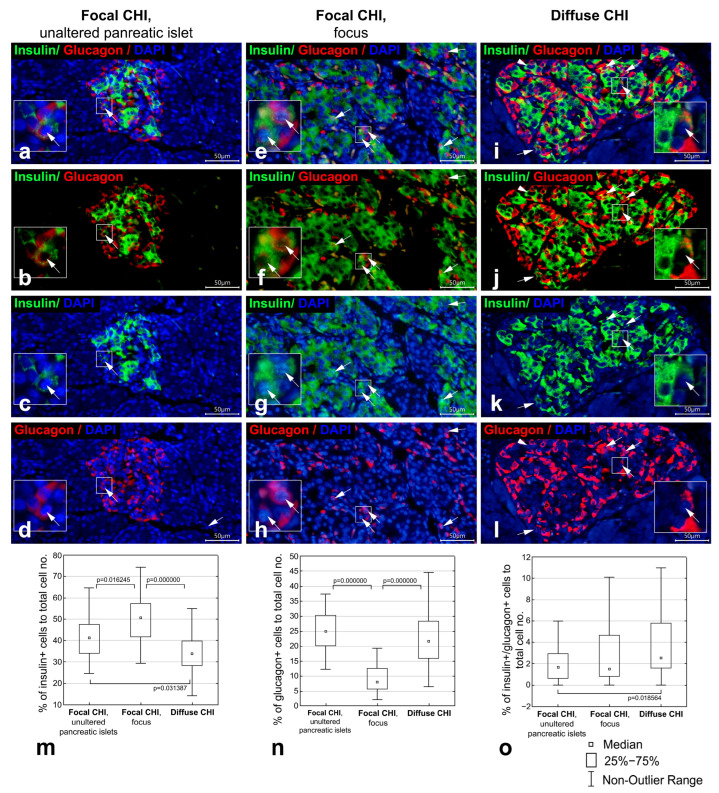
Pancreatic sections from infants with focal CHI (case 2; (**a**–**h**)) and diffuse CHI (case 6; (**i**–**l**)); double immunofluorescent staining for insulin (green) and glucagon (red); nuclei are counterstained with DAPI (blue). (**a**–**d**) unaltered pancreatic islet with intermixed β- and α-cells. (**e**–**h**) focus of adenomatous hyperplasia in which β-cells prevail and α-cells are less abundant. (**i**–**l**) diffuse CHI, enlarged pancreatic islet with intermixed β- and α-cells. Bi-hormonal insulin+/glucagon+ cells (indicated by arrows) are more abundant within the focus (**e**–**h**) and in diffuse CHI (**i**–**l**) than in unaltered pancreatic islets (**a**–**d**). Arrowhead indicates a β-cell with enlarged nucleus. Particulars marked by squares are enlarged in insets. Scale bar 50 μm. (**m**–**o**) Boxplots illustrating the proportion of insulin+ (**m**), glucagon+ (**n**), and insulin+/glucagon+ (**o**) cells in unaltered pancreatic islets, within the focus, and in diffuse CHI; significant differences (nonparametric Kruskal–Wallis ANOVA test with post hoc multiple comparisons of mean ranks of all pairs of groups) are marked by square brackets and *p*-values.

In areas outside the focus, pancreatic islets had unaltered morphology in patients with focal CHI (cases 1–4). The islets were rounded, oval or elongated in shape, with intermixed β- and α-cells (Figure 1a–d) or with centrally located β-cells surrounded by α-cells. Co-localization of insulin and glucagon was seen in a small number of cells (Figure 1a–d).

**Figure 2:**
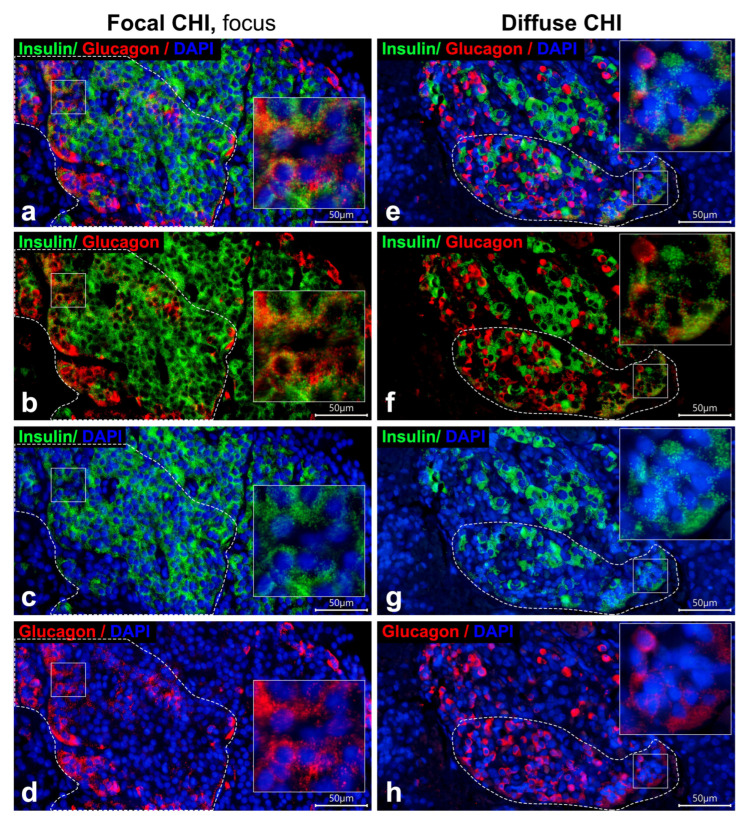
Pancreatic sections from infants with focal CHI (case 5; (**a**–**d**)) and diffuse CHI (case 11; (**e**–**h**)). Double immunofluorescent staining for insulin (green) and glucagon (red). Nuclei are counterstained with DAPI (blue). In some areas (outlined by dotted line) of the focus (**a**–**d**) and within pancreatic islets in diffuse CHI (**e**–**h**), numerous cells simultaneously containing sparse insulin+ and glucagon+ granules are seen. Particulars marked by squares are enlarged in insets. Scale bar 50 μm.

The morphology of the focus varied across cases of focal CHI. In cases 1–3, the focus consisted of a single node of adenomatous hyperplasia exhibiting trabecular or nested architecture formed by endocrine cells, occasionally with duct-like structures among them; no acini were identified (Figure 1e–h). In cases 4 and 5, the focus comprised hypertrophic, irregularly shaped pancreatic islets distributed within the acinar parenchyma (Figure 2a–d). Generally, within the focus, β-cells predominated, while α-cells were less frequent (Figure 1e–h and Figure 2a–d). Compared to unaltered pancreatic islets (Figure 1a–d), the number of α-cells in the focus appeared reduced (Figure 1e–h and Figure 2a–d). Bi-hormonal insulin+/glucagon+ cells were identified within the focus in all cases and were more abundant than in unaltered islets (Figure 1e–h and Figure 2a–d). Bi-hormonal cells were mostly isolated among other endocrine cells; however, in cases 3, 4, and 5, they were observed in small clusters within the focus (Figure 2a–d). Isolated bi-hormonal cells contained densely packed immunoreactive granules, whereas in clustered cells, the granules appeared more sparsely and diffusely distributed.

In diffuse CHI, islet architecture resembled that of unaltered pancreatic islets. The islets had rounded, oval, or elongated shapes and displayed either an intermix of β- and α-cells or centrally located β-cells with peripherally located α-cells (Figure 1i–l and Figure 2e–h). The size of the islets in diffuse CHI appeared larger than that of unaltered pancreatic islets, and the majority of islets in diffuse CHI contained β-cells with enlarged nuclei (Figure 1i–l). Bi-hormonal cells were more numerous than in unaltered islets (Figure 1i–l and Figure 2e–h). In two cases of diffuse CHI (cases 6 and 11), we found clusters of bi-hormonal cells containing sparse insulin+ and glucagon+ granules within some pancreatic islets (Figure 2e–h). These areas were morphologically similar to those observed within the focus in three cases of focal CHI.

To confirm our qualitative observations, we employed quantitative morphometric analysis to compare the distribution of islet cell types (insulin+, glucagon+, and insulin+/glucagon+) in different forms of CHI with unaltered pancreatic islets. The raw measurements and calculated morphometric parameters are shown in Table S1.

In focal CHI, the focus consisted predominantly of endocrine cells, resulting in significantly larger regions of interest (areas with stained endocrine cells) compared to unaltered islets and to islets in diffuse CHI (Table 3). In diffuse CHI, pancreatic islets were also significantly larger than unaltered islets (Table 3).

Compared with unaltered pancreatic islets, cell density was lower within the focus of focal CHI and in diffuse CHI (Table 3).

Within the focus, the proportion of insulin+ cells was increased (Figure 1m, Table 3), while the proportion of glucagon+ cells was decreased (Figure 1n, Table 3) compared to unaltered pancreatic islets. In diffuse CHI, the proportion of insulin+ β-cells was decreased compared to unaltered islets (Figure 1m, Table 3), whereas the proportion of glucagon+ α-cells did not differ between these groups (Figure 1n, Table 3). Bi-hormonal insulin+/glucagon+ cells represented 1.68%, 1.52%, and 2.55% of the total cell number and 3.8%, 3.0%, and 6.6% of the total insulin+ cell population in unaltered pancreatic islets, within the focus, and in diffuse CHI, respectively (Table 3). In diffuse CHI, the proportion of insulin+/glucagon+ of the total cell number was increased compared to unaltered pancreatic islets (Figure 1o, Table 3). Although insulin+/glucagon+ cells were more frequently observed within the focus than in unaltered pancreatic islets, this difference was not statistically significant (Figure 1o, Table 3).

### 3.2. Co-Localization of PDX1 with Insulin and Glucagon in Diffuse and Focal CHI

**Figure 3:**
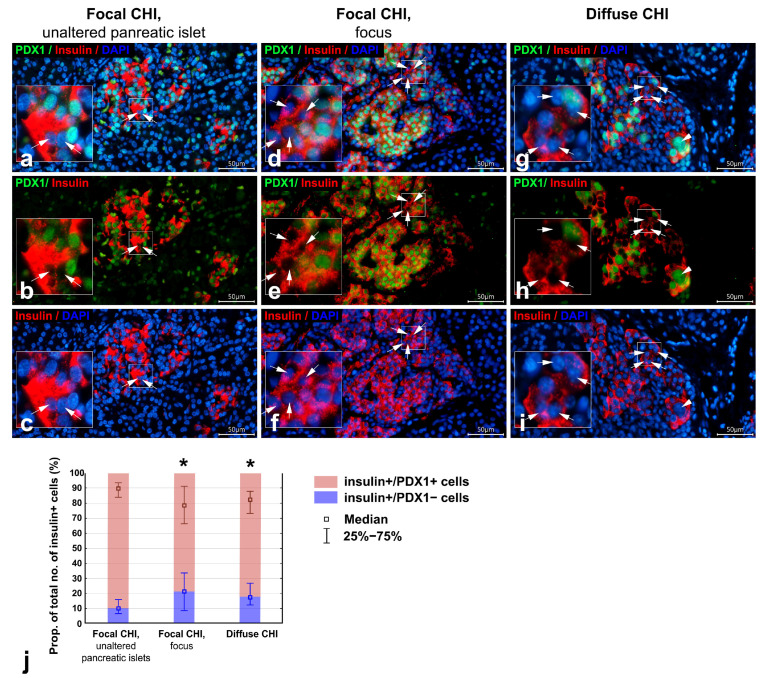
Pancreatic sections from infants with focal CHI and diffuse CHI; double immunofluorescent staining for PDX1 (green) and insulin (red); nuclei are counterstained with DAPI (blue). (**a**–**c**) case 1, focal CHI, unaltered pancreatic islet; (**d**–**f**) case 5, focal CHI, focus; (**g**–**i**) case 7, diffuse CHI. In the unaltered pancreatic islets (**a**–**c**), positive staining for PDX1 is observed in the majority of insulin+ cells, with only rare cells lacking PDX1 (insulin+/PDX1− cells; arrows). Within the focus (**d**–**f**) and in diffuse CHI (**g**–**i**), numerous insulin+ cells lacking PDX1 staining (insulin+/PDX1− cells; arrows) are present. Particulars marked by squares are enlarged in insets. Scale bar 50 μm. Histograms in (**j**) illustrate a significant increase in the proportion of insulin+/PDX1− cells with a corresponding decrease in the proportion of insulin+/PDX1+ cells among all insulin+ cells within the focus and in diffuse CHI compared to unaltered pancreatic islets (nonparametric Kruskal–Wallis ANOVA test with post hoc multiple comparisons of mean ranks of all pairs of groups; significant differences are indicated by asterisks).

We examined the distribution of the β-cell–specific transcription factor PDX1 in different forms of CHI. PDX1 staining was detected in the nuclei of the majority of insulin+ β-cells (insulin+/PDX1+) across all samples (Figure 3a–i). However, some insulin+ cells lacked PDX1 expression (insulin+/PDX1−). In the focus and in islets from diffuse CHI, the proportion of insulin+/PDX1− cells appeared to be higher than in unaltered pancreatic islets (Figure 3d–i).

**Figure 4:**
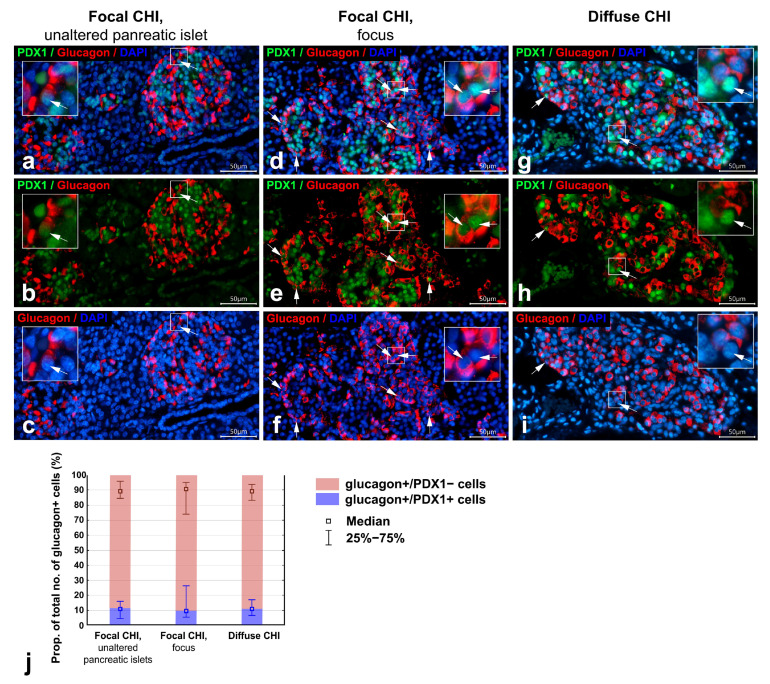
Pancreatic sections from infants with focal CHI and diffuse CHI; double immunofluorescent staining for PDX1 (green) and glucagon (red); nuclei are counterstained with DAPI (blue). (**a**–**c**) case 1, focal CHI, unaltered pancreatic islet; (**d**–**f**) case 5, focal CHI, focus; (**g**–**i**) case 7, diffuse CHI. In the unaltered pancreatic islets (**a**–**c**), within the focus (**d**–**f**), and in diffuse CHI (**g**–**i**), the majority of glucagon+ cells are negative for PDX1; cells double positive for glucagon and PDX1 (glucagon+/PDX1+ cells, arrows) are very rare. Particulars marked by squares are enlarged in insets. Scale bar 50 μm. Histograms in (**j**) illustrate the proportion of glucagon+/PDX1+ and glucagon+/PDX1− cells among all glucagon+ cells in unaltered pancreatic islets, within the focus, and in diffuse CHI. No significant group differences were found (nonparametric Kruskal–Wallis ANOVA test with post hoc multiple comparisons of mean ranks of all pairs of groups).

PDX1 staining was absent in most glucagon+ α-cells (glucagon+/PDX1−) (Figure 4a–i). Only rare glucagon+ cells with PDX1-positive nuclei (glucagon+/PDX1+) were observed, both in unaltered pancreatic islets and in diffuse CHI, as well as within the focus (Figure 4a–i).

The raw measurements and calculated morphometric parameters for the PDX1 distribution in insulin+ and glucagon+ cells are shown in Tables S2 and S3. Quantitative morphometric analysis revealed that the proportion of insulin+/PDX1+ cells among all insulin+ cells was decreased, while the proportion of insulin+/PDX1− cells was increased in diffuse CHI and within the focus compared to unaltered pancreatic islets (Table 3, Figure 3j). Conversely, no significant differences were observed in the proportions of glucagon+/PDX1+ cells and glucagon+/PDX1− cells among all glucagon+ cells between groups (Table 3, Figure 4j).

## 4. Discussion

Monogenic forms of CHI are generally considered to involve predominantly β-cell alterations [1,3,5,6,7,27,28]. However, in our opinion, these forms of CHI are more accurately understood as a disease of the entire pancreatic islet. Genetic alterations not only cause β-cells hypersecretion, but also shift the cellular balance between the different islet cell types. In healthy individuals, intercellular interaction should repress excessive activity of some cell types and enhance function of others. If the inhibitory function of antagonist cells is insufficient, proliferation of these cells may occur. Therefore, changes of a balance between β-cells and their antipode—α-cells, and molecular aspects of this process are of great interest.

We have investigated pancreatic samples from five children with focal CHI and six children with diffuse CHI and obtained morphological evidences of alterations of islet cell phenotypes associated with CHI.

In focal CHI, mutations in the *ABCC8* and *KCNJ11* genes in combination with a specific loss of maternal alleles in the imprinted chromosome region 11p15, lead to abnormal proliferation and morphogenesis of β-cells, resulting in the formation of the hyperplastic focus of hyperfunctioning β-cells [1,14]. As expected, the regions of interest (areas containing stained endocrine cells) within the focus were larger than unaltered pancreatic islets.

In diffuse CHI, the size of pancreatic islets was also significantly larger than that of unaltered pancreatic islets. This finding contradicts a previous study that reported no differences in islet size between diffuse CHI and normal tissue [29]. The discrepancy is likely due to differences in morphometric methodology. In our study, we measured the area of individual islets and compared median values between groups, whereas Goudswaard et al. [29] assessed islet size distribution within a defined tissue area. The observed differences may also be influenced by the individual variability in islet morphology and the small number of investigated cases both in our study (six cases) and the previous report [29] (five cases). Therefore, larger studies are necessary to more precisely evaluate islet size in diffuse CHI.

In both the focus and diffuse CHI, pancreatic islets were larger and exhibited reduced cell density compared to unaltered pancreatic islets. This could be explained by hypertrophy of hyperfunctioning islet cells, which may have undergone shrinkage during subsequent histological processing.

Consistent with previous morphological reports [8,9,29], focal CHI is characterized by a higher proportion of insulin+ β-cells and a lower proportion of glucagon+ α-cells within the focus, compared to unaltered tissue and diffuse CHI. The reduced number of glucagon+ α-cells within the focus may result either from expansion of β-cell mass alone or from a combination of β-cell expansion and decreased α-cell formation. Studies on pancreatic embryogenesis indicate that proliferation predominantly occurs at the progenitor stage, prior to hormone expression, after which endocrine lineages diverge and differentiate [30,31,32]. Preferential activation of the β-cell differentiation program may suppress α-cell differentiation, contributing to the cellular imbalance observed within the focus in focal CHI.

The finding of a significant decrease in the proportion of insulin+ β-cells in diffuse CHI compared with unaltered pancreatic islets contradicts previous reports, which detected no significant difference between these groups [8,9,29]. In the present study, insulin+ cells and bi-hormonal insulin+/glucagon+ cells were quantified as distinct cell types. In addition, we evaluated the relative proportions of different endocrine cell types within individual pancreatic islets. This approach minimizes the impact of factors such as cell size, islet distribution density, and the abundance of other pancreatic cell types on the measured proportions of specific cell types, in contrast to approaches that estimate the total mass of each cell type across the whole pancreatic sample. These methodological differences may account for discrepancies compared to previous findings.

The results obtained in humans with diffuse CHI also differ from those reported in laboratory animal model injected with human induced stem cells. In this model of CHI, the number of β-cells was significantly increased compared to the control group [33]. This once again supports the view of Salisbury et al. that animal models of CHI, even those based on the injection of human cells, do not yet fully reproduce all aspects of the human disease [15].

Bi-hormonal cells producing insulin and glucagon are present in the human pancreas during normal prenatal development [32,34,35,36] and may represent differentiating endocrine cells. In infants and children, bi-hormonal insulin+/glucagon+ cells constitute less than 2% of total islet cells, as reported in Figure 4a of the study of Hahm and colleagues [37], which aligns with our findings in unaltered islets of patients with focal CHI. In adults, bi-hormonal cells are very rare, but their frequency increases with age [37] and in pathological conditions such as type 2 diabetes [24,25,38]. A low level of insulin has also been observed in glucagon+ α-cells of insulin-deficient islets of individuals with longstanding type 1 diabetes [21]. Similar increases in the proportion of bi-hormonal cells have been reported in animal models of diabetes [17,24]. Many authors propose that the appearance of bi-hormonal cells in diabetes is related to the degranulation of β-cells, their dedifferentiation into immature phenotypes, and subsequent transdifferentiation into α-cells [17,18,20,23,25]. Alternatively, bi-hormonal cells may represent α-cells expressing insulin as a compensatory response to insulin insufficiency [21], or differentiating β-cells that fail to attain a mature phenotype [39,40,41].

Salisbury et al. reported insulin+/glucagon+ co-positive cells in only two of ten diffuse CHI samples [15]. In contrast, our study detected such cells in all examined cases. The proportion of insulin+/glucagon+ cells was significantly higher in diffuse CHI compared to unaltered islets and focus in focal CHI. Although the functional significance of bi-hormonal insulin+/glucagon+ cells in CHI remains uncertain, their increased prevalence in diffuse CHI may reflect altered islet cell identity and enhanced cellular plasticity. We hypothesized that genetically driven insulin hypersecretion in diffuse CHI might lead to β-cell degranulation followed by dedifferentiation and transdifferentiation, as previously demonstrated in β-cells exposed to hyperglycemia in diabetes models [23,24,25].

We found that focal lesions contained visually more bi-hormonal cells than unaltered pancreatic islets; however, this difference was not statistically significant. This pattern suggests that dedifferentiation or transdifferentiation of islet cells, especially β-cells, may be less prominent in focal CHI than in diffuse CHI. The lack of a significant difference between focal lesions and unaltered islets likely reflects the markedly increased proportion of insulin+ cells within the focus and the substantial morphological heterogeneity characteristic of focal CHI.

Given the evidence supporting potential transdifferentiation between β- and α-cells [19,20,38,42,43], we performed double immunofluorescent labeling to analyze the distribution of cells co-expressing PDX1 with insulin or glucagon. PDX1 is a key transcription factor regulating β-cell differentiation, maturation, and survival [44,45]. In the developing human pancreas, PDX1 expression is detected in epithelial progenitors and newly formed β-cells [34], whereas in the postnatal period it is predominantly restricted to β-cells [22,46]. Consistent with these data, in all samples examined in our study, PDX1 staining was mainly observed in the nuclei of insulin+ β-cells, while the majority of glucagon+ α-cells were PDX1-negative.

The most notable finding was the presence of a large number of insulin+ β-cells lacking PDX1 expression within the focus and in diffuse CHI. In unaltered pancreatic islets, insulin+/PDX1− cells accounted for approximately 10.1%, aligning with recent data by Granlund et al. (2024) [46], which reported about 6% of PDX1-negative β-cells in normal islets. The occasional absence of PDX1 in β-cells has also been observed in infants with diffuse CHI [15]. Our quantitative analysis demonstrated that the proportion of insulin+/PDX1− cells was nearly doubled in both the focus and diffuse CHI compared with unaltered islets.

A decrease in PDX1 expression could represent an early step in the initiation of the apoptotic pathway in β-cells. However, the proportion of insulin+/PDX1− cells among all insulin+ cells within the focus and in diffuse CHI observed in the present study (17.66% in focus and 21.54% in diffuse CHI) was significantly higher than the percentage of β-cell apoptosis reported in other studies (<5% apoptotic β-cells in both types of CHI) [15,47].

We suggest that a subset of cells is capable of synthesizing insulin through a PDX1-independent mechanism. These may represent pre-existing β-cells that, for unknown reasons, have lost PDX1 expression and acquired less mature phenotype. This suggestion is supported by previous findings showing increased expression of other immaturity markers, such as NeuroD1 and Nkx2.2, in islet cells of infants and children with CHI [13,15]. Alternatively, these cells could be bi-hormonal, co-expressing insulin and other hormones, such as dedifferentiating or transdifferentiating cells with an insulin+/glucagon+/PDX1− phenotype. The proportion of insulin+/glucagon+ cells among all insulin+ cells was 3.88% in unaltered pancreatic islets, 3.00% within the focus, and 6.61% in diffuse CHI. Therefore, approximately one third of insulin+/PDX1− cells may be bi-hormonal cells co-expressing insulin and glucagon. Other types of islet cells, such as δ- or PP-cells that have acquired the ability to express insulin, may also contribute to insulin+/PDX1− cells.

A small subset of glucagon+ α-cells showed positive staining for PDX1 in all cases, including unaltered islets. Previous studies have reported the presence of occasional glucagon+/PDX1+ cells in the human pancreas during early prenatal development [34] and in adults [46]. The proportion of these cells has been shown to increase in individuals with type 1 diabetes [21,22]. However, in our study, the proportion of glucagon+ cells expressing PDX1 within the focus and in diffuse CHI did not differ from that in unaltered pancreatic islets. This suggests that the metabolic conditions in CHI may have a less pronounced effect on α-cells compared with diabetes.

In summary, we observed an increased proportion of β-cells lacking PDX in both focus and diffuse CHI, along with an increased proportion of bi-hormonal insulin+/glucagon+ cells in diffuse CHI. These results suggest that β-cells may dedifferentiate into less mature phenotypes or transdifferentiate into α-cells in CHI. However, such phenotypic alterations of β-cells appear to be less pronounced in focal CHI, as the proportion of bi-hormonal cells in the focal lesion was not significantly different from that in unaltered islets.

Remarkably, these changes resemble those reported in diabetes, where β-cell degranulation and dedifferentiation into immature phenotypes occur in response to metabolic stress caused by hyperglycemia. In contrast, CHI represents the opposite metabolic state—chronic hyperinsulinism and hypoglycemia—yet the β-cells show comparable morphological alterations. This paradox suggests that β-cell degranulation, whether triggered by excessive stimulation or by glucose toxicity, may converge on a common pathway of cellular dedifferentiation.

In CHI, these changes appear to be largely confined to β-cells, as α-cell morphology and marker expression remain unaffected. This selective vulnerability implies that β-cells are the primary targets of stress induced by genetically driven insulin hypersecretion. Overall, our findings highlight a potentially universal adaptive mechanism of β-cell stress: under both hyper- and hypoglycemic conditions, β-cells may lose their mature phenotype and revert toward a less differentiated state.

## 5. Conclusions

This study provides morphological evidence of altered islet cell phenotypes in CHI. We identified two main abnormalities: (1) an increased proportion of insulin+/PDX1− β-cells, and (2) a higher number of bi-hormonal insulin+/glucagon+ cells in diffuse CHI. These findings indicate that a subset of β-cells in CHI loses typical markers of maturity or begins to co-express hormones characteristic of other endocrine cell types. We propose that these changes result from genetically driven insulin hypersecretion, which leads to β-cell degranulation and subsequent dedifferentiation into less mature phenotypes. Our findings provide a structural basis for future functional studies aimed at evaluating the role of islet cell plasticity in the pathogenesis and treatment of CHI.

## 6. Limitations

We have evaluated the distribution of bi-hormonal cells and the transcription factor PDX1 in infants and children with CHI and suggested that alterations in β-cell phenotype may occur in this pathology. These alterations are less evident in focal CHI because the proportion of bi-hormonal cells within the focus did not differ from that in unaffected tissue.

Only five cases of focal CHI and six cases of diffuse CHI were analyzed, as severe pharmacoresistant CHI is rare. We used pancreatic tissue located outside the focal lesion as a comparison group. While this tissue is genetically unaffected, it may still be influenced by the altered metabolic conditions associated with CHI.

We used only a limited panel of markers: PDX1 as a marker of β-cell maturity and insulin/glucagon co-localization as an indicator of potential β- to α-cell conversion. Co-localization of these markers was analyzed using fluorescent microscopy on 4 μm thick sections, which minimizes the possibility of misidentification due to overlap of cells from different layers. However, confocal microscopy may enhance the accuracy of co-localization assessment.

Future morphological studies with a larger number of samples, including healthy controls, as well as the application of additional markers of β-cell maturity and functional state, are necessary to better understand alterations in the β-cell phenotype in CHI.

Functional metabolic assays were not performed in this study, as such tests are not ethically feasible in infants with CHI and are not part of routine clinical evaluation. Given the rarity of resected pancreatic tissue and the clinical severity of CHI, our findings provide a structural basis for future functional studies aimed at evaluating the role of islet cell plasticity in the pathogenesis and treatment of CHI.

## Figures and Tables

**Table 1:** Characteristics of the investigated cases of CHI.

Case No.	Sex	Age of Onset	Age at Surgery, Month	Genetics	Minimal Glucose Level mmol/L	Maximal Insulin Level at the Time of Hypoglycemia, μU/mL	Treatment, Maximum Doses Used (Assessed Retrospectively)	PET ^18^F-DOPA Scan Results	Histological Form of CHI, Group
1	F	2nd day of life	12	Heterozygous paternal R1436G in ABCC8	1.2	18.0	Diazoxide 7 mg/kg/day, Octreotide 30 mcg/kg/day with no improvement	Focal uptake	Focal CHI, unaltered pancreatic islets Focal CHI, focus
2	M	3rd day of life	22	c.A680G:p.E227G heterozygous paternal in KCNJ11	1.4	15.2	Diazoxide 20 mg/kg/day, Octreotide 34 mcg/kg/day with no improvement	Focal uptake	Focal CHI, unaltered pancreatic islets Focal CHI, focus
3	F	1st day of life	7	R705X heterozygous paternal in ABCC8	1.8	38.0	Diazoxide 16 mg/kg/day, Octreotide 20 mcg/kg/day with no improvement	Focal uptake	Focal CHI, unaltered pancreatic islets Focal CHI, focus
4	F	2nd day of life	5	c.G1332T: p.Q444H heterozygous paternal in ABCC8	0.7	45.0	Diazoxide 5.5 mg/kg/day—discontinued due to side effects, Octreotide 20 mcg/kg/day with no improvement	Focal uptake	Focal CHI, unaltered pancreatic islets Focal CHI, focus
5	M	1st day of life	5	c 3330-13G>A heterozygous paternal in ABCC8	0.8	3.24	Diazoxide 10.3 mg/kg/day with no improvement	Focal uptake	Focal CHI
6	F	1st day of life	7	c.1037C>T:p.A346V homozygous in KCNJ11	0.2	6.5	Diazoxide 11.9 mg/kg/day, Octreotide 10 mcg/kg/day with no improvement	Focal uptake	Diffuse CHI
7	F	1st day of life	3	C87R homozygous in KCNJ11	1.0	32.0	Diazoxide 16 mg/kg/day, Octreotide 33 mcg/kg/day with no improvement	Diffuse uptake	Diffuse CHI
8	F	13 months	19	c.1361 1363dupCGG heterozygous in GCK	0.6	28.0	Diazoxide 18 mg/kg/day, Octreotide 20 mcg/kg/day with no improvement	Diffuse uptake	Diffuse CHI
9	F	1st day of life	26	Q444H/R1250X compound heterozygous in ABCC8	1.9	50.0	Diazoxide 15 mg/kg/day, Octreotide 20 mcg/kg/day with no improvement	Diffuse uptake	Diffuse CHI
10	F	1st day of life	2	c.405dupG:p.R136 AfsX 5 homozygous in KCNJ11	1.1	9.3	Diazoxide 16 mg/kg/day, Octreotide 10 mcg/kg/day + continuous dextrose infusion 8.8 mg/kg/min with no improvement	Diffuse uptake	Diffuse CHI
11	F	1st day of life	2	c.C387T:p.A129A heterozygous in HNF4A	0.1	24	High volume infusion, dextrose requirement rate 22.5 mg/kg/min, Diazoxide was contraindicated, Octreotide maximum dose 11.25 mg/kg/min, Glucagon infusion with no improvement, severe hypoglycemia and high infusion rate prompted surgical treatment	Diffuse uptake	Diffuse CHI

**Table 2:** Characteristics of the used primary and secondary antibodies.

Antibodies	Company	Cat.#/RRID	Dilutions
Mouse monoclonal antibodies to insulin, clone INS04; INS05	Thermo Fisher Scientific Inc., Fremont, CA, USA	MS-1379-P/ RRID:AB_62834	1:100
Rabbit polyclonal antibodies to glucagon	Thermo Fisher Scientific Inc., Regensburg, Germany	PA5-13442/ RRID:AB_2107206	1:50
Mouse monoclonal antibodies to glucagon, clone K79bB10	Sigma, St. Louis, MO, USA	G2654/ RRID:AB_259852	1:4000
Rabbit monoclonal antibodies to PDX1, clone EP-139	Epitomics, Burlingame, CA, USA	AC-0131C	1:100
IFluor™594 conjugated goat anti-mouse IgG	Huabio, Hangzhou, China	HA1126	1:500
IFluor™488 conjugated goat anti-rabbit IgG	Huabio, Hangzhou, China	HA1121	1:500
IFluor™488 conjugated goat anti-mouse IgG	Huabio, Hangzhou, China	HA1125	1:500
IFluor™594 conjugated goat anti-rabbit IgG	Huabio, Hangzhou, China	HA1122	1:500

**Table 3:** The values of the investigated morphometric parameters in the unaltered pancreatic islets and within the focus in focal CHI, and in diffuse CHI.

Parameter	Focal CHI, Unaltered Pancreatic Islets	Focal CHI, Focus	Diffuse CHI
Islet area, μm^2^	9328.14 (5578.41–12,798.92) (*n* = 40)	36,727.73 * (23,276.46–55,095.92) (*n* = 50)	15,661.71 * (12,302.61–22,310.03) (*n* = 60)
Cell density (the no. of DAPI-positive nuclei per μm^2^ of islet area)	0.016 (0.013–0.019) (*n* = 40)	0.013 * (0.011–0.014) (*n* = 50)	0.011 * (0.010–0.012) (*n* = 60)
Proportion of insulin+ cells of total cell no. (%)	41.37 (34.22–47.82) (*n* = 40)	50.83 * (41.86–57.59) (*n* = 50)	34.09 * (28.57–39.64) (*n* = 60)
Proportion of glucagon+ cells of total cell no. (%)	24.94 (20.05–30.39) (*n* = 40)	8.07 * (5.60–12.65) (*n* = 50)	21.65 (16.07–28.32) (*n* = 60)
Proportion of insulin+/glucagon+ cells of total cell no. (%)	1.68 (0.58–2.97) (*n* = 40)	1.52 (0.83–4.69) (*n* = 50)	2.55 * (1.60–5.83) (*n* = 60)
Proportion of insulin+/glucagon+ cells of total no. of insulin+ cells (%)	3.86 (1.60–6. 85)	3.00 (1.67–7.69)	6.61 (3.30–17.48) *
Proportion of insulin+/PDX1+ cells of total no. of insulin+ cells (%)	89.90 (84.01–93.41) (*n* = 44)	78.46 * (66.43–91.30) (*n* = 50)	82.34 * (73.08–87.85) (*n* = 60)
Proportion of insulin+/PDX1− cells of total no. of insulin+ cells (%)	10.10 (6.59–15.99) (*n* = 44)	21.54 * (8.70–33.57) (*n* = 50)	17.66 * (12.15–26.92) (*n* = 60)
Proportion of glucagon+/PDX1+ cells of total no. of glucagon+ cells (%)	10.87 (4.26–15.56) (*n* = 47)	9.41 (4.92–26.19) (*n* = 50)	10.75 (6.38–16.98) (*n* = 63)
Proportion of glucagon+/PDX1− cells of total no. of glucagon+ cells (%)	89.13 (84.44–95.74) (*n* = 47)	90.59 (73.81–95.08) (*n* = 50)	89.25 (83.02–93.62) (*n* = 63)

Values are presented as the median and the upper and lower quartile values (Me (q1–q3)) and the number of observations (*n*); «*»—Significantly differed from the unaltered pancreatic islets.

## Data Availability

Data presented in this study are contained within this article and Supplementary Materials (Tables S1–S3).

## Supplementary Materials

The following supporting information can be downloaded at: https://www.mdpi.com/article/10.3390/life16010018/s1, Table S1: Morphometric parameters obtained from sections double-stained with antibodies to insulin and glucagon; Table S2: Morphometric parameters obtained from sections double-stained with antibodies to insulin and PDX1; Table S3: Morphometric parameters obtained from sections double-stained with antibodies to glucagon and PDX1.
